# Range-wide genetic analysis of an endangered bumble bee (*Bombus affinis,* Hymenoptera: Apidae) reveals population structure, isolation by distance, and low colony abundance

**DOI:** 10.1093/jisesa/ieae041

**Published:** 2024-04-03

**Authors:** John M Mola, Ian S Pearse, Michelle L Boone, Elaine Evans, Mark J Hepner, Robert P Jean, Jade M Kochanski, Cale Nordmeyer, Erik Runquist, Tamara A Smith, James P Strange, Jay Watson, Jonathan B U Koch

**Affiliations:** Department of Forest and Rangeland Stewardship, Warner College of Natural Resources, Colorado State University, Fort Collins, CO, USA; Graduate Degree Program in Ecology, Colorado State University, Fort Collins, CO, USA; Graduate Degree Program in Ecology, Colorado State University, Fort Collins, CO, USA; U.S. Geological Survey, Fort Collins Science Center, Fort Collins, CO, USA; Department of Entomology, University of Minnesota, Saint Paul, MN, USA; Department of Entomology, University of Minnesota, Saint Paul, MN, USA; Metamorphic Ecological Research and Consulting, LLC, Alonzaville, VA, USA; Environmental Solutions and Innovations, Inc., Indianapolis, IN, USA; Department of Integrative Biology, University of Wisconsin–Madison, Madison, WI, USA; Conservation Department, Minnesota Zoo, Apple Valley, MN, USA; Conservation Department, Minnesota Zoo, Apple Valley, MN, USA; U.S. Fish and Wildlife Service, Minnesota–Wisconsin Ecological Services Field Office, Bloomington, MN, USA; Department of Entomology, The Ohio State University, Columbus, OH, USA; Wisconsin Department of Natural Resources, Green Bay, WI, USA; U.S. Department of Agriculture, Agricultural Research Service, Pollinating Insect Research Unit, Logan, UT, USA

**Keywords:** endangered species, conservation genetics, bumble bee, pollinator decline, bee, conservation, species recovery, *Bombus affinis*, rusty patched bumble bee

## Abstract

Declines in bumble bee species range and abundances are documented across multiple continents and have prompted the need for research to aid species recovery and conservation. The rusty patched bumble bee (*Bombus affinis*) is the first federally listed bumble bee species in North America. We conducted a range-wide population genetics study of *B. affinis* from across all extant conservation units to inform conservation efforts. To understand the species’ vulnerability and help establish recovery targets, we examined population structure, patterns of genetic diversity, and population differentiation. Additionally, we conducted a site-level analysis of colony abundance to inform prioritizing areas for conservation, translocation, and other recovery actions. We find substantial evidence of population structuring along an east-to-west gradient. Putative populations show evidence of isolation by distance, high inbreeding coefficients, and a range-wide male diploidy rate of ~15%. Our results suggest the Appalachians represent a genetically distinct cluster with high levels of private alleles and substantial differentiation from the rest of the extant range. Site-level analyses suggest low colony abundance estimates for *B. affinis* compared to similar datasets of stable, co-occurring species. These results lend genetic support to trends from observational studies, suggesting that *B. affinis* has undergone a recent decline and exhibit substantial spatial structure. The low colony abundances observed here suggest caution in overinterpreting the stability of populations even where *B. affinis* is reliably detected interannually. These results help delineate informed management units, provide context for the potential risks of translocation programs, and help set clear recovery targets for this and other threatened bumble bee species.

## Introduction

Declining trends in insect abundance and diversity have increased awareness of the need to enact conservation programs aimed at preserving insect species and their habitats (Wagner 2020). Primary among these has been the recognition of declining bumble bee populations (Cameron et al. 2011, Cameron and Sadd 2020). Due to the higher quality of data on bumble bees compared to other insect taxa, their decline has been argued as a harbinger for other insect species more widely (Goulson and Nicholls 2016). Like other species, loss and degradation of habitat, introduced pathogens, pesticides, and climate change, as well as interactive and additive effects, factor into bumble bee declines (Cameron and Sadd 2020).

Informed conservation programs require genetic data to illuminate what cannot otherwise be seen from simple counts of species or measurements of morphological characteristics alone (Funk et al. 2019). The availability of genetic data for conservation programs helps us to understand the health of particular bumble bee populations, compare bumble bee population genetics to that of relatively stable species, and set target metrics for “genetic health” for listed species. This information also helps to delineate genetic management units, evaluates the risks and benefits of activities such as species reintroductions or augmentations, and aids the appropriate allocation of limited resources. Genetic information serves as a necessary first step in determining priorities and setting baselines for species-focused conservation, which sets the stage for more targeted ecological studies to hone species recovery and habitat restoration programs.

Genetic factors may play a role in bumble bee population declines, with small effective population sizes and reduced gene flow from fragmented habitats resulting in reduced viability of populations. Inbreeding and small effective population sizes may be particularly disadvantageous for bumble bees and other insects in the order Hymenoptera because, in these insects, heterozygosity determines sexual development (Zayed and Packer 2005). Thus, when AR is low, high numbers of diploid males can be produced, which increases genetic load and decreases reproductive fitness (Zayed et al. 2003). Prior studies of bumble bee population genetics in eastern North America suggest that stable species have limited evidence of population structure and relatively high colony abundance compared to declining species (Cameron et al. 2011). However, the population genetics of the first bumble bee species to be listed as federally endangered through the US Endangered Species Act (U.S. Fish and Wildlife Service 2017a, 2017b), the rusty patched bumble bee (*Bombus affinis*, Cresson), has not been studied.

**Fig. 1.**
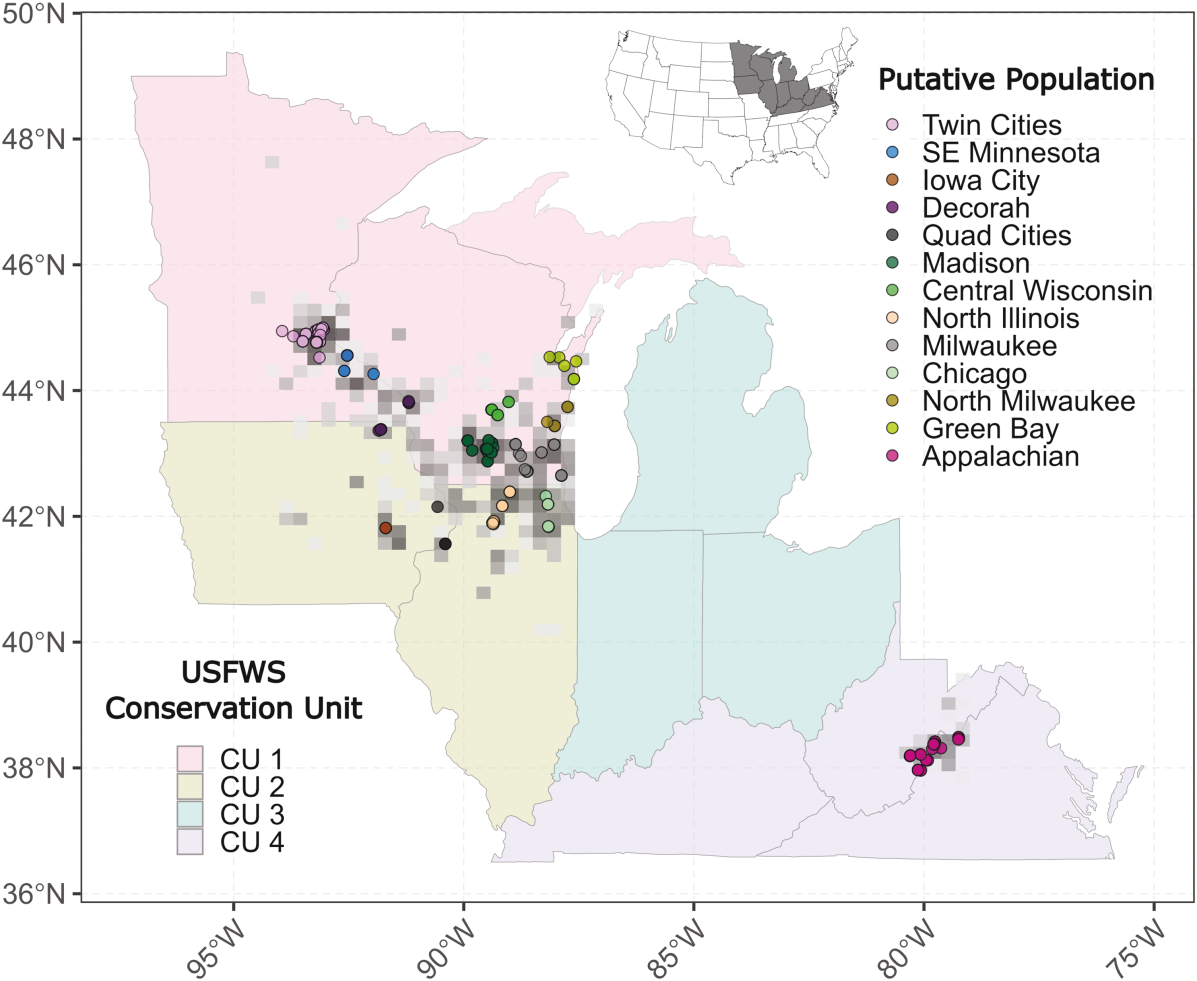
Map of the study area showing locations of individual specimens of *Bombus affinis* collected from the field in 2020 and 2021 colored by putative 100-km populations (circles) relative to the density of all US Fish and Wildlife Service *B. affinis* records from 2015 through 2021 (gray overlay). Background fill colors of states represent the recovery plan CU boundaries (US Fish and Wildlife Service 2021). For visual simplicity, CU 5 and states without records from 2015 to 2021 are not shown. The inset map provides regional context within the United States.

*Bombus affinis* began declining in the late 1990s and has been lost from an estimated ~70%–90% of its historical range (Giles and Ascher 2006, Colla et al. 2012, Szymanski et al. 2016). The species was once broadly distributed across the northeastern United States and southeastern Canada but now is largely restricted to the upper midwestern United States and parts of the Appalachian Mountains (Fig. 1). The rusty patched bumble bee represents a broader conservation push toward understanding the causes of the decline of North American bees and finding recovery solutions.

Basic population genetic information would inform the conservation of *B. affinis* in several meaningful ways. First, population genetics can help measure the genetic health of particular extant populations and develop target metrics for genetic health, a population attribute specifically named in the *B. affinis* Recovery Plan (US Fish and Wildlife Service 2021). Identifying populations that suffer from high levels of inbreeding and low levels of heterozygosity can inform management actions to increase habitat connectivity and gene flow among populations (Lozier and Zayed 2016). Second, understanding the overall population structure of this bee can inform the delineation of management units within larger, established conservation units (CU) (Fig. 1) and guide conservation actions within those units, as genetically disparate populations may warrant additional attention or distinct management actions. Uncovering any broad-scale population structure of this bee is critical prior to potential translocation efforts by identifying populations genetically compatible with management goals, whether those be preserving population distinctiveness or facilitating admixture (Smith et al. 2020). Third, genetic information is integral to counting the reproductive units (colonies) of bumble bees. This is because the reproductive unit for bumble bees is a colony, whereas the typical observation of a bumble bee is that it is a nonreproductive worker foraging outside the nest. Through genotyping, individuals observed foraging can be assigned to colonies, and then colony abundance may be estimated via genetic mark-recapture (Darvill et al. 2004, Mola et al. 2020). Populations at sites with very low colony abundance may be susceptible to extinction due to stochastic demographic effects and may warrant management actions to avoid major disturbances that could exacerbate those effects. All told, there is an urgent need for foundational population genetics work on *B. affinis* to inform recovery programs, minimize potential impacts to *B. affinis*, and enable future, more targeted research.

In this study, we present the results of a first-ever range-wide population genetics study with the federally endangered rusty-patched bumble bee (*B. affinis*). We report standard genetic measurements, based on microsatellite markers, to answer the following broad questions: (i) what is the broad-scale population structuring of *B. affinis*? and (ii) what are the patterns of population genetic diversity and differentiation across its extant range? Additionally, we use genetic mark-recapture analysis at the site level to answer a third question: (iii) what are the common colony abundances of *B. affinis* within sites, and how do they compare to other co-occurring *Bombus* species?

## Materials and Methods

### Specimen Collection

We collected nonlethal tarsal samples from *B. affinis* across its extant range (Fig. 1). Because the species is uncommon and tarsal clipping is restricted to only some federal scientific recovery permit holders, the collection of specimens represented an effort beyond the ability of any single team. Instead, we coordinated with several teams that were conducting parallel projects on the species. Due to this coordinated collection and variation in the rate of detection of *B. affinis* among sites, our sample sizes and relative effort are imbalanced, but the spatial spread well represents the extant range of the species (Fig. 1), allowing accurate examination of our primary questions. Field collections were made of males or females as they were encountered, and care was taken to avoid queens or early season workers following best practices (Mola et al. 2021) and within permit conditions and appropriate handling agreements between permit holders and USFWS. In 2020 and 2021, we collected tarsal samples from a total of 498 individual *B. affinis*. After basic filtering for quality controls (known date, sex, accurate latitude, and longitude), we had 470 unique specimens available for study collected between 17 June and 20 September, although 96.5% of specimens were collected in July or August. Of these specimens, 46 were from 3 known colonies (e.g., Boone et al. 2022) and were not included in the downstream analysis for consistency across our sampling range. We describe the sample sizes associated with each analysis after appropriate filtering in the relevant methods sections below. Data and code generated in this study are available via the Dryad Digital Repository (Mola et al. 2024).

Because our specimens were collected opportunistically across the species’ range without a priori assumptions of genetic populations, we assigned putative sites and populations to our samples prior to genetic analysis. To assign specimens to putative sites, we conducted hierarchical spatial clustering using the “hclust” function within r::stats (all analysis conducted in R version 4.2.1 unless otherwise indicated; R Core Team 2022) using the complete method. We then defined sites and populations at buffers of 10 km and 100 km, respectively, using the “cutree” function. The 10 km site-level buffer is chosen to be in alignment with the scale considered within the *B. affinis* recovery plan (U.S. Fish and Wildlife Service 2021), and the 100 km population-level buffer is chosen to represent a value well outside of expected dispersal distances for bumble bees (Lepais et al. 2010, Mola and Williams 2019). Hereafter, we refer to the 10 km clusters as “sites” and the 100 km clustering as “populations” or “putative populations.” This procedure resulted in 59 sites (Supplementary Fig. S1) and 13 populations (Fig. 1). Downstream analysis may use only a subset of these sites or populations due to filtering, as indicated in each section.

### DNA Extraction and Genotyping

Total genomic DNA was extracted from the tarsal tissue samples using a Quick DNA Miniprep Kit (Zymo Research) following the manufacturer’s protocol. Extracted DNA was subjected to 2 multiplex reactions with 15 5ʹ dye-labeled primers previously identified in the literature as suitable for a wide range of *Bombus* species (Estoup et al. 1995, 1996, Funk et al. 2006, Stolle et al. 2009). In multiplex reaction 1, the following loci were PCR amplified (dye label chemistry identified in parenthesis): B124 (6-FAM), BTERN01 (VIC), BT28 (VIC), BT10 (NED), B96 (PET), BT30 (PET), and BTMS0081 (PET). In multiplex reaction 2, the following loci were amplified: BTMS0066 (6-FAM), BTMS0083 (6-FAM), B126 (VIC), BTMS0062 (VIC), BTERN02 (NED), BTMS0086 (NED), BL13 (PET), and BTMS0059 (PET). PCR reactions were performed using the following methods: 1 µl template DNA, 1× Promega reaction buffer (Madison, WI), 1.4 mM MgCl_2_, 0.6 mM dNTP mixture, 0.2–0.4 µM primer, 0.001 mg BSA, 0.4 units Taq Polymerase (Promega, Madison, WI, USA). PCR conditions for both multiplex reactions were one period at 95 °C for 3:30 min, 30 cycles at 95 °C for 30 s, and annealing temperature at 55/58 °C (multiplex reaction 1 = 55 °C, multiplex reaction 2 = 58 °C) for 1:15 min, 72 °C for 45 s, and a final extension period at 72 °C for 15 min. Each multiplex PCR representing the dye-labeled conditions was separated along with a dye-labeled size standard (GeneScan 500 LIZ dye size standard, Applied Biosystems, ThermoFisher Scientific) on an Applied Biosystems 3730xl automatic sequencer at the Utah State University Center for Integrated Biosystems. Fragment length polymorphisms resulting from the sequencer were then scored using Geneious Prime 2021.0.1 (Kearse et al. 2012). The remaining extracted DNA and tissue samples are stored at the USDA ARS Pollinating Insect Research Unit in Logan, UT, for future research endeavors such as high-throughput genomic sequencing.

### Hardy–Weinberg Equilibrium, Linkage Disequilibrium, and Null Alleles

To ensure the quality of our genotype data before subsequent analysis, we tested for deviations from Hardy–Weinberg equilibrium (HWE), linkage disequilibrium (LD), and the presence of null alleles. To test for HWE, we used the “hw.test” function from the r::adegenet package (Jombart 2008), and corrected for multiple testing using both the chi-squared and Monte Carlo permutation tests. We found that loci were out of HWE globally, but only due to differences among subpopulations. We tested for LD using the “ia” function from the r::poppr package (Kamvar et al. 2014). Significant LD was not detected, and no consistent LD was found in pairwise comparisons, suggesting that correlation among markers is unlikely to be a source of error in subsequent analyses. The frequency of null alleles was inferred with the “null.all” function from r::PopGenReport (Adamack and Gruber 2014). All observed frequencies were below 0.15 and well within reported ranges for microsatellite studies, and they were unlikely to cause bias in downstream population structure analysis (Dakin and Avise 2004).

### Colony Assignment

To assign individuals to colonies for downstream analysis, full siblings were assigned to putative colonies within COLONY v2.0 (Jones and Wang 2010). To determine microsatellite genotyping error (Supplemental Table S1), 48 samples were subjected to a second round of PCR, fragment analysis, and genotyping at the target loci. From these loci, we estimated the mean error rate per locus (*e*_*l*_), which has the capacity to identify error-prone loci, and is calculated as follows: *e*_*l*_ = *ml*/*nt*, where *ml* is the number of single-locus genotypes that include allelic mismatch, and *nt* is the number of replicated single-locus genotypes (Pompanon et al. 2005). We ran COLONY using the full-likelihood method for haplodiploid species, assuming monogamy for both males and females (Owen and Whidden 2013). See data release for complete settings (Mola et al. 2024). Because running COLONY with all samples together yielded inconsistent results or “impossible” pairings (e.g., siblings separated by hundreds of km or across years), likely due to low levels of allelic diversity within the species, we ran COLONY with 308 females (i.e., all collected females passing initial quality filtering as described in *Specimen Collection* section) in batches split between populations and year. We then filtered to retain all putative assignments at a cluster probability threshold of 0.80. Preliminary analysis with repeated runs of COLONY deemed a more restrictive inclusion threshold unnecessary. To avoid pseudo-replication in the subsequent analysis of genetic structure, diversity, and population differentiation, we randomly retained one sibling per colony. After selecting a single random individual from each colony, 231 females were included in our analyses of population genetic structure, genetic diversity, and differentiation (Table 1). For our analysis of site-level colony abundance (see *Site-Level Colony Abundance* section), we retained all 308 females, which is necessary to calculate colony abundance accurately.

**Table 1.** Summary statistics of population genetic diversity metrics for the geographically clustered 100-km putative populations of *Bombus affinis.* Populations are arranged from west (top) to east (bottom) according to their central longitude. Values for expected heterozygosity and inbreeding coefficient represent the mean across all loci. Compare to Fig. 3 for results of statistically significant tests

100-km population	Number of specimens	Private alleles	Rarefied AR	Expected heterozygosity (*H*_*E*_)	Inbreeding coefficient (*F*_IS_)
Twin Cities	60	22	4.12	0.70	0.19
SE Minnesota	8	6	3.40	0.63	0.15
Iowa City	4	0	3.31	0.61	0.11
Decorah	8	0	3.90	0.68	0.36
Quad Cities	11	1	3.73	0.67	0.18
Madison	35	1	4.05	0.68	0.16
Central Wisconsin	7	0	3.88	0.73	0.60
North Illinois	19	1	3.99	0.69	0.32
Milwaukee	40	0	4.08	0.69	0.17
Chicago	7	1	3.80	0.69	0.26
North Milwaukee	4	0	4.08	0.76	0.31
Green Bay	7	0	3.52	0.61	−0.03
Appalachian	21	16	3.96	0.68	0.04

### Population Genetic Structure

To examine population genetic structure, we first used the clustering method within the software STRUCTURE version 2.3.4 (Pritchard et al. 2000). STRUCTURE assumes individuals are comprised of K unknown populations to which fractional genotypes can be assigned. We ran STRUCTURE using the admixture model with 20,000 burn-in steps and 100,000 samples. We ran 10 iterations for each K value, with K ranging from 1 to 10. Next, to determine the optimal K (i.e., number of genetic clusters), we examined the probability of the data as described by Evanno et al. (2005) using the Δ*K* method from the “evannoMethodStructure” function in package r::pophelper (Francis 2017). To account for variation among individual STRUCTURE runs, we averaged admixture proportions over the10 replicates for the best K using “alignK” and then “mergeQ” within r::pophelper (Francis 2017). To spatially visualize average K-assignments within each putative population, we took the mean of cluster assignments across all individuals within a putative population.

We also conducted a Discriminant Analysis of Principal Components (DAPC) using the r::adegenet package and the “dapc” function (Jombart 2008). DAPC can complement STRUCTURE as it allows us to visualize variation among populations in a method that is robust to the assumptions of HWE. DAPC detects clustering in genetic data by applying a principal component analysis (PCA) that maximizes the between-group variance and minimizes the within-group variance (Jombart 2008). The number of clusters was determined using Bayesian Information Criterion values. To identify the optimal range of principal components (PCs) to retain for discriminant analysis, the “a-score” function from r::adegenet was used. The optimal number of PCs to retain was determined by comparing the proportion of successful reassignments to values obtained with random groups, thus avoiding overfitting of the data (Jombart 2008).

### Genetic Diversity

We calculated common measures of genetic diversity to characterize *B. affinis* populations using putative populations and the clusters as determined from the output of STRUCTURE. The genetic clusters should be examined with some caution, as 2 clusters (“Twin Cities” and “Appalachian”) consist of only a single 100 km population each. However, this approach allowed for the most reasonable and management-relevant comparison of measures across populations without issues of excessive comparisons or low sample sizes. For each putative population and STRUCTURE-assigned cluster, we calculated private AR using “private_alleles” within r::poppr (Kamvar et al. 2014), rarefied allelic richness (AR) using “allelic.richness” within r::hierfstat, and measures of observed and expected heterozygosity (*H*_o_, *H*_e_) and the coefficient of inbreeding (*F*_IS_) using “basic.stats” in r::hierfstat (Goudet 2005).

For each of He, AR, and *F*_IS_ we fit a series of linear mixed effects models using r::lme4 (Bates et al. 2015) by putative population or genetic cluster (i.e., Twin Cities, Central, and Appalachian; *see below*). Each model fits with the genetic diversity measurement of interest (He, AR, and *F*_IS_) as a function of the fixed effect of putative population or cluster and locus as a random effect (Soro et al. 2017). We tested the model for statistical significance using likelihood ratio tests of the focal model against a null model with the fixed effect removed. We calculated marginal and conditional *R*^2^ using “r.squaredGLMM” within r::MuMIn (Barton and Barton 2015). Finally, to determine significant pairwise differences among populations or clusters, we conducted post hoc Tukey’s pairwise significance tests using the “glht” function within the r::multcomp package (Hothorn et al. 2016).

We also estimated the presence of diploid males within each population. The presence of diploid males within haplodiploid Hymenoptera suggests evidence of inbreeding (Zayed 2004). For males at each 100 km putative population, we documented the number of heterozygous loci. We had 115 males which were used for estimating diploidy and inbreeding. In microsatellite analysis, males should all be homozygous, but the presence of heterozygosity suggests diploidy. Because this can arise from simple genotyping error, we only count an individual as diploid when 3 or more loci are heterozygous (Darvill et al. 2006).

### Population Differentiation

To test for evidence of isolation by distance (IBD), we calculated the pairwise fixation index (*F*_ST_) of all 100 km populations. Pairwise *F*_ST_ was calculated using the Weir and Cockerham (1984) method within package r::hierfstat (Goudet 2005). We then tested for IBD by conducting a Mantel test using the “mantel” function within the r::vegan package (Oksanen et al. 2022) and the “spearman” method, as our data show nonlinear patterns of differentiation. To conduct this test, we used a matrix of genetic distance (pairwise *F*_ST_) and geographic distance between the putative population centroids.

### Site-Level Colony Abundance

To describe colony abundance at sites with sufficient specimens we report the estimated number of colonies using a genetic mark-recapture procedure. To determine the estimated number of colonies, we used the output of program COLONY (*described above*) at all 10 km radius sites that contained at least 9 specimens. This cutoff point represented a sample size at which we could reliably run mark-recapture models (*see below*); no siblings are observed beyond this distance and represent sites with repeatedly observed *B. affinis* foragers year-to-year as reported from local researchers or managers. For each site and year, we report the number of individuals genotyped at that site (*N*_gen_). We then estimated the number of unique colonies observed at each site using the output of COLONY after filtering (*N*_col_). Next, based on mark-recapture logic, we calculated the maximum likelihood number of colonies (*N*_ML_) at each site using the R package “capwire” and the 2-innate rate model (Goulson et al. 2010, Mola et al. 2020). We ran the bootstrapping procedure using 1,000 resamples to determine the upper and lower 95% confidence interval. We then report the proportion of detected colonies (*N*_det_) by dividing *N*_col_ by *N*_ML_. Finally, to place our results within a broader context, we compared the proportion of unique colonies (*N*_unique_ = *N*_col_/*N*_gen_) at all sites to data from Cameron et al. (2011) for 2 stable co-occurring species (*B. impatiens*, *B. bimaculatus*) and one at-risk species (*B. pensylvanicus*) using the same modeling procedure from that study. To determine if *B. affinis* workers represent fewer colonies than expected compared to sites with stable species, we fit a generalized linear model of *N*_unique_ at all sites with >1 individual as a function of species with a quasibionomial error structure and used a Tukey multiple comparisons test to test for significant differences between the proportion of unique colonies for *B. affinis* and the other 3 species.

Lastly, we also report effective population size (*N*_*e*_) at these same sites with ≥9 specimens. We report the estimate from COLONY’s random mating model using the estimated sibship structure (Wang 2009). We report results from the random mating model as it has fewer assumptions about HWE and sensitivities to sample size. Additionally, preliminary analysis showed no substantial differences in results using the random or nonrandom model. COLONY was rerun to calculate these *N*_*e*_ estimates at the 10 km sites with ≥9 specimens, and no changes in sibling assignment were observed compared to the full run (described above).

## Results

### Microsatellite Assessment

The microsatellite loci BTERN01, BT28, BT30, BTMS0081, BTMS0066, B126, BTMS0086, BL13, and BTMS0059 demonstrated an error rate of 5.10% or less. B96, BTMS0083, and BTMS0062 demonstrated an error rate between 8.51% and 10.63%. Finally, B124, BT10, and BTERN02 demonstrated an error rate between 27.77% and 98.93% (Supplementary Table S1). Based on Estoup et al. (1996), the expected motif of B96 is 2 base pairs; however, our genotype assessment of *B. affinis* demonstrated evidence for a 3 base pair motif. BTMS0083 and BTMS0062 are highly polymorphic each with 37 polymorphic alleles, potentially increasing the likelihood of genotyping error. Removal of the 2 loci with >90% error (B124 and BT10) found a significant and positive correlation between the mean error rate and number of alleles per locus (Kendall Correlation Test: Tau = 0.49, *z* = 2.24, *P* = 0.02). Thus, based on the genotyping error rates estimated from the PCR amplified loci, we elected to retain the following loci for downstream analyses: BTERN01, BT28, BT30, BTMS0081, BTMS0066, B126, BTMS0086, BL13, BTMS0059, B96, BTMS0083, BTMS0062, and BTERN02 (Supplementary Table S1). Although BTERN02 had an error rate of 27.77%, the inclusion of this locus had no impact on downstream analysis of colony assignment or population genetic measurements, so the decision to retain it for simplicity was made.

### Population Genetic Structure

**Fig. 2.**
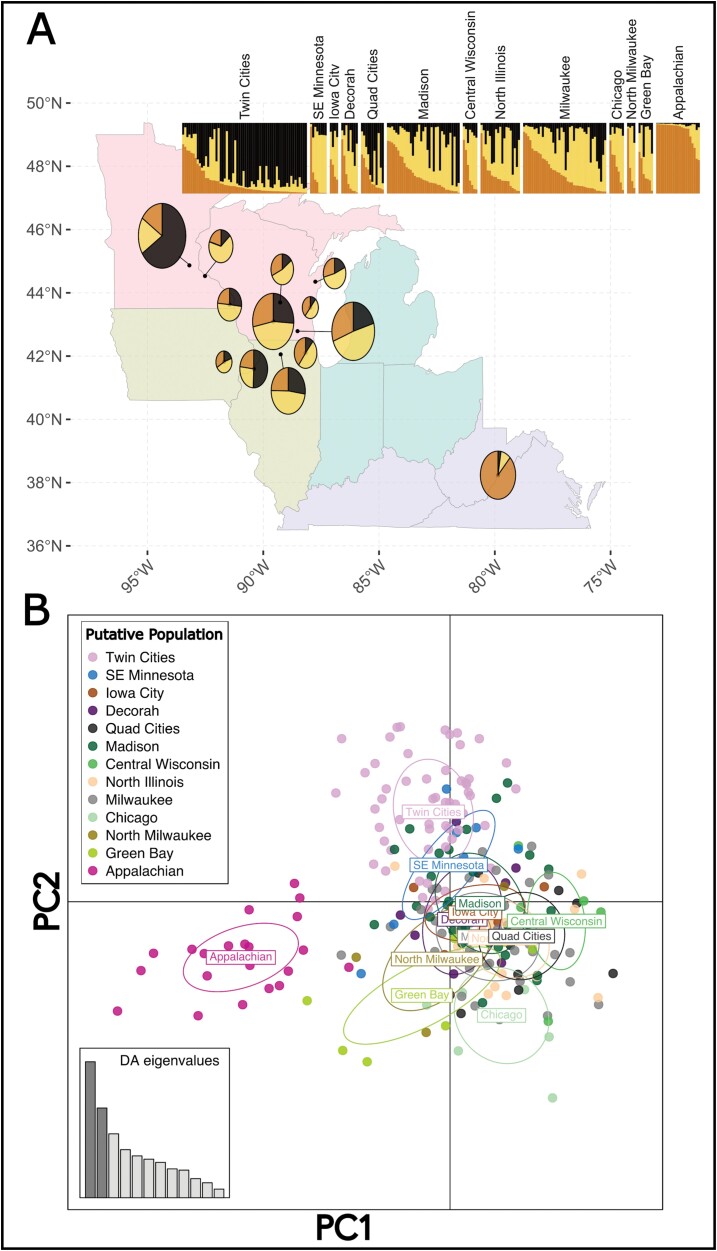
Results for *Bombus affinis* populations of A) STRUCTURE analysis showing clustering of putative populations to genetic clusters (*K* = 3). The size of the circle represents the number of individuals sampled per putative population (circle size is nonlinearly scaled for visual clarity). The size of each pie slice represents the average genetic assignment of all individuals in each population to one of K populations. Inset bar plot where each bar represents an individual and each color represents identity to one of K genetic clusters. Sites are separated by vertical white bars and ordered by longitude (west to east; left to right). Full sample size informative for putative populations can be obtained from Table 1. B) Discriminant Analysis of Principal Components (DAPC) scatterplot showing differentiation across the first 2 PCs. Circles are colored by putative population for visual comparison to Fig. 1 and to demonstrate the divergence of Appalachian and Twin Cities populations from the remaining species’ range among PCs 1 and 2, respectively.

Multiple lines of evidence suggest that there is a population genetic structure within the extant range of *B. affinis* with 3 distinct clusters (Fig. 2). First, the most explanatory number of genetic clusters as determined from the Evanno test (i.e., highest Δ*K*) was *K* = 3, with substantially less explanatory power gained by including additional clusters. Second, the results of STRUCTURE at *K* = 3 suggest the 3 most reasonable clusters for *B. affinis* to be a northwestern cluster (hereafter “Twin Cities”), a geographically large central cluster (“Central”), and the Appalachians (“Appalachian”; Fig. 2A). Finally, analysis with DAPC was largely congruent with these assigned clusters (Fig. 2B). Similar to the Evanno test, the unsupervised *k*-means clustering resulted in a most parsimonious *K* value of 3. DAPC analysis using *K* = 3 retained 60 PCs, explaining about 88.4% of the variance among 12 discriminant eigenvalues (Fig. 2B). The scatterplot of the first 2 PCs shows a separation of individuals from the Appalachian cluster separated out along PC1 and Twin Cities cluster specimens separated out from the Central cluster predominantly along PC2 (Fig. 2B).

### Genetic Diversity

**Fig. 3.**
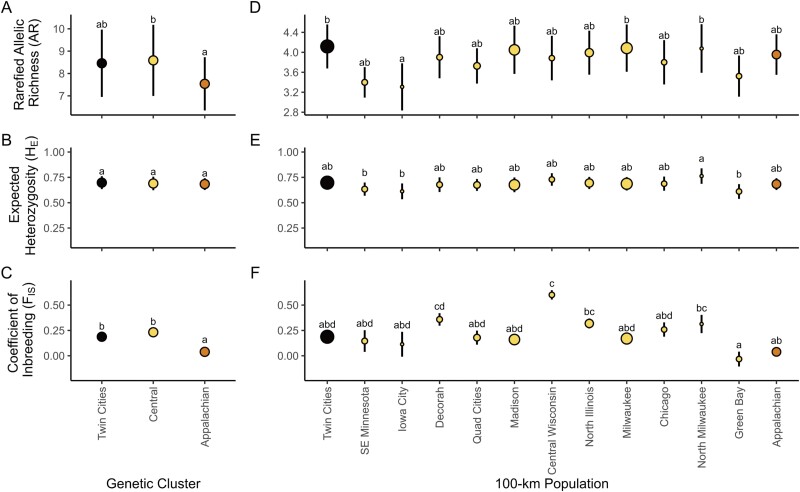
Plots of genetic diversity measurements (mean ± SE across loci) for *Bombus affinis* at the genetic cluster level following STRUCTURE A–C) and geographically assigned 100 km population level D–F). A and D) rarefied AR. Note the different *y*-axis scales due to differences in minimum sample size for rarefaction procedure at different scales, B and E) expected heterozygosity, and C and F) inbreeding coefficient. Statistical significance is shown using shared lettering for any groups that are not significantly different following a Tukey’s post hoc significance test on linear mixed effects models with loci as a random effect. In plots D–F), point size represents a number of specimens.

Analysis of genetic diversity across the different putative populations showed differences among those, although caution is warranted in assessing this due to low sample sizes in some populations (Table 1; Fig. 3). Analysis at the cluster level showed that H_e_ was not differentiated among clusters (*R*^2^_*m*_ = 0.007, *R*^2^_*c*_ = 0.914, *P* = 0.855). However, among clusters, there was evidence of differences in rarefied AR (*R*^2^_*m*_ = 0.009, *R*^2^_*c*_ = 0.951, *P* = 0.046) and *F*_IS_ (*R*^2^_*m*_ = 0.366, *R*^2^_*c*_ = 0.563, *P* < 0.001) with the Appalachian cluster having lower AR and *F*_IS_ values compared to the Central and Twin Cities cluster (Fig. 3). There was no statistical difference in rarefied AR or F_IS_ between Central or Twin Cities clusters (Fig. 3).

Our analysis of male diploidy suggests that out of 115 individuals, 18 of them were diploid for a range-wide rate of ~15% male diploidy. Putative populations ranged from 0% to 100% diploidy in males (Table 2).

**Table 2.** Sample sizes of males were collected across 100-km putative populations of *Bombus affinis* with a number of diploid males as determined from a threshold of 3 heterozygous loci. Populations are arranged from west (top) to east (bottom) according to their central longitude

100 km population	*N* males	*N* diploid males	Proportion diploid males
Twin Cities	50	8	0.16
SE Minnesota	6	2	0.33
Iowa City	1	0	0.00
Decorah	4	0	0.00
Quad Cities	6	0	0.00
Madison	1	0	0.00
Central Wisconsin	0	0	—
North Illinois	1	1	1.00
Milwaukee	14	1	0.07
Chicago	3	3	1.00
North Milwaukee	3	0	0.00
Green Bay	0	0	—
Appalachian	26	3	0.12

### Population Differentiation

**Fig. 4.**
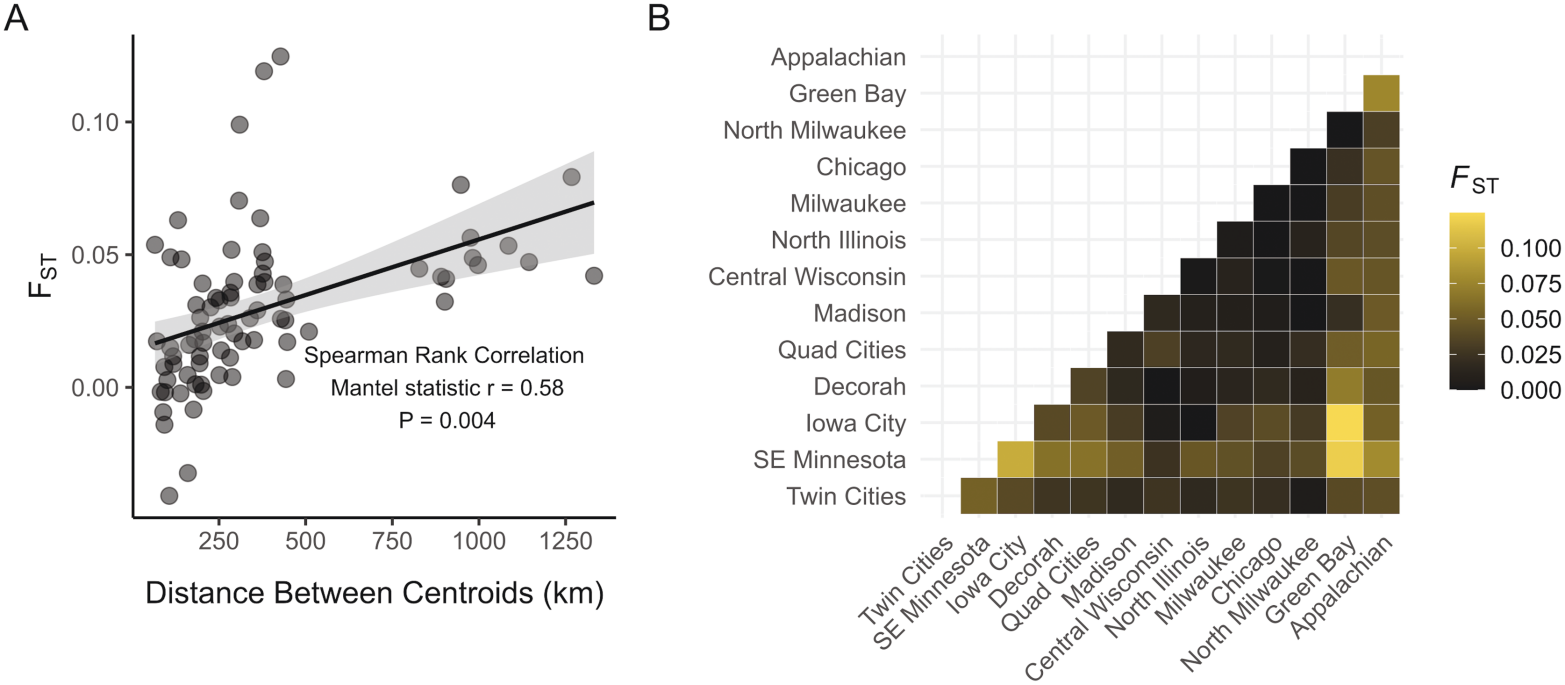
Pairwise linear fixation index (*F*_ST_) for *Bombus affinis* populations across A) geographic distance (kilometer) with results of Mantel test using Spearman’s rank correlation method and B) matrix of *F*_ST_ values by putative population. Shading in A) represents 95% confidence interval.

We detected strong genetic differentiation relative to geographic distance (i.e., IBD) across the range of *B. affinis* (Mantel test: *r* = 0.5848, *P* = 0.004; Fig. 4A). There was substantial pairwise population differentiation (i.e., *F*_ST_) across the species range among putative populations (Fig. 4B).

### Site-level Colony Abundance

**Fig. 5.**
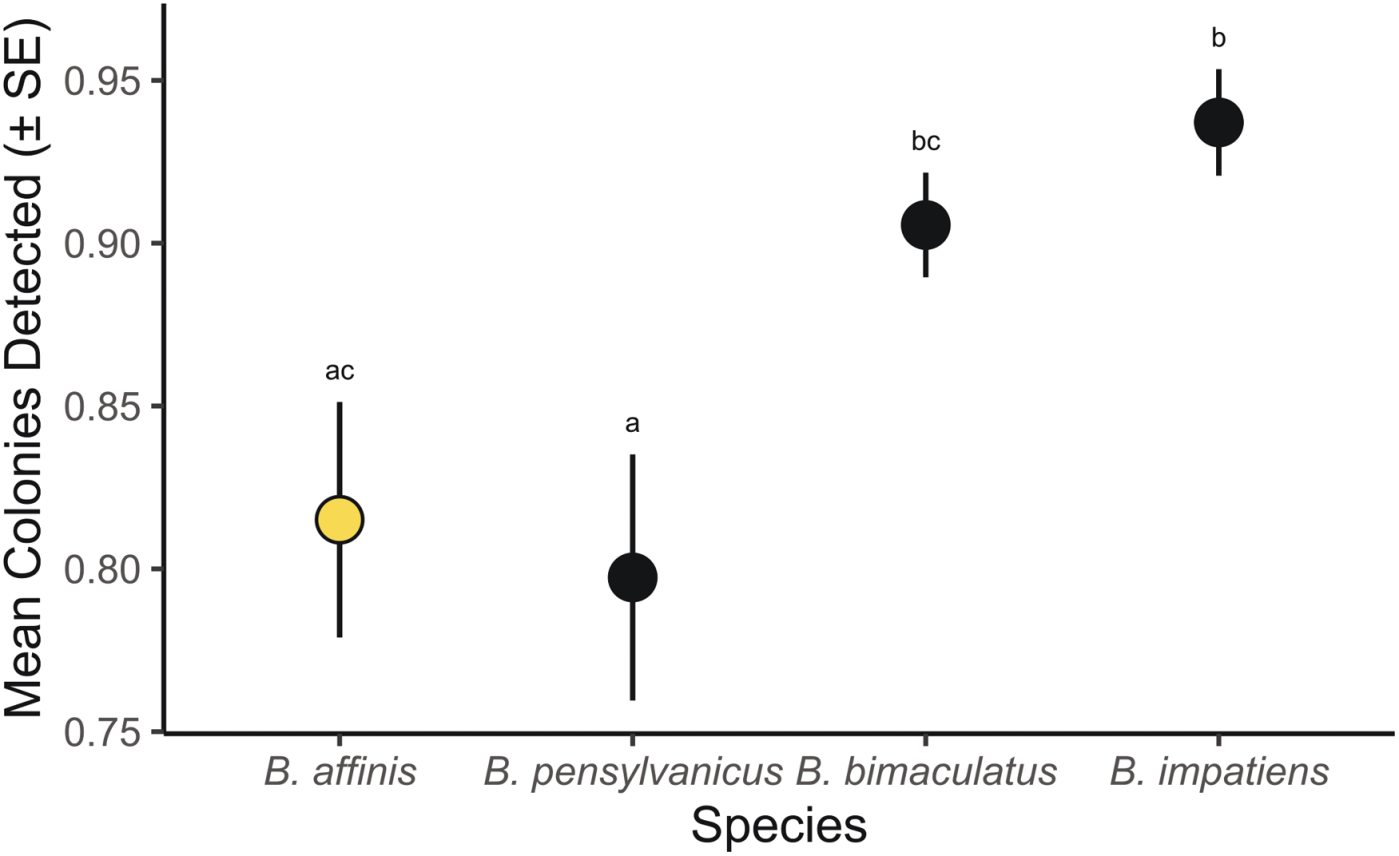
Mean (±SE) proportion of unique colonies (*N*_col_/*N*_gen_) for *Bombus affinis* (from this study) and *Bombus pensylvanicus* (declining), *Bombus bimaculatus* (stable), and *Bombus impatiens* (stable; data for 3 comparative Eastern species from Cameron et al. 2011). Species with shared lettering are not significantly different from each other from a Tukey’s multiple comparisons test on the quasibinomial linear model.

We found substantial variation in the estimated abundance of colonies among sites (Table 3). Variation in the number of observed colonies (*N*_col_) is likely in part due to variation in the number of individuals sampled among sites. However, the variation in the proportion of detected colonies (*N*_det_) was also substantial, suggesting a large variation in the true number of colonies (estimated here using mark-recapture as *N*_ML_) among sites (Table 3). Compared to stable species, we found a significantly lower estimate of unique colonies (*N*_unique_) for *B. affinis* compared to *B. impatiens* (*B. affinis* mean = 0.815, *B. impatiens* mean = 0.937, *P* = 0.01; i.e., for any collection of 100 *B. affinis* or *B. impatiens* workers we would expect them to be from ~81 vs. ~93 colonies, respectively) and marginally significantly lower estimates in comparison to *B. bimaculatus* (*B. affinis* mean = 0.815, *B. bimaculatus* mean = 0.906, *P* = 0.09), and found a similar proportion of unique colonies as another at-risk species, *B. pensylvanicus* (*B. affinis* mean = 0.815, *B. pensylvanicus* mean = 0.797, *P* = 0.97; Fig. 5). Estimates of effective population size (*N*_*e*_) per site ranged from 5 to 48 and generally were correlated with estimates of colony number (Pearson’s product-moment correlation = 0.85, df = 9, *P* = 0.001; Table 3).

**Table 3.** Number of genotyped samples (*N*_geno_), unique colonies detected from COLONY (*N*_col_), proportion of unique colonies (*N*_unique_), maximum likelihood estimate of colonies (*N*_ML_), and proportion of detected colonies of *B. affinis* from 10-km sites with at least 9 specimens in a year (*N*_det_). Dashes for the Appalachian site represent a case where the maximum likelihood procedure cannot be run due to no sibling pairs being detected. Effective population size (*N*_*e*_) is reported from the random mating model output of COLONY. CI = confidence interval

10 km site, y	*N*_geno_	*N*_col_	*N*_unique_ (*N*_col_/*N*_geno_)	*N*_ML_ (95% CI)	*N*_det_ (*N*_col_/*N*_ML_)	*N*_e_ (95% CI)
Illiniwek, 2021	11	10	0.91	56 (23–1,000)	0.18	41 (21–129)
Nachusa Grasslands, 2021	15	13	0.87	70 (34–1,000)	0.19	43 (24–94)
Capitol Springs, 2021	9	8	0.89	36 (11–1,000)	0.22	32 (15–118)
Foxglove Savannah, 2021	13	10	0.77	38 (19–1,000)	0.26	16 (8–35)
Minnesota Zoo, 2021	19	15	0.79	41 (19–174)	0.37	48 (30–88)
Cherokee, 2021	11	8	0.73	20 (8–56)	0.40	31 (16–87)
Turtle Valley, 2021	42	27	0.64	66 (50–126)	0.41	38 (25–63)
Minnesota Zoo, 2020	18	9	0.50	16 (9–29)	0.56	10 (5–24)
Downtown Minnesota, 2020	10	3	0.30	5 (3–6)	0.60	5 (2–20)
Seed Savings Exchange, 2021	9	4	0.44	5 (3–9)	0.80	6 (2–21)
Hamline, 2021	12	4	0.33	4 (3–6)	1.00	6 (3–20)
Appalachian, 2021	9	9	1.00	—	—	32 (15–115)

## Discussion

For the first time, we document the population genetics of the federally listed rusty-patched bumble bee (*B. affinis*). We find patterns consistent with expectations for an endangered species. Namely, a spatially structured range exhibiting patterns of IBD and small population sizes (i.e., colony abundance). Although it has been known for some time that *B. affinis* has declined in abundance and has been lost from ~70% to 90% of its historical range (Giles and Ascher 2006, Colla et al. 2012, Szymanski et al. 2016), the results presented here provide insight into how to effectively manage remaining populations and prioritize or guide conservation and research efforts.

### Population Genetic Structure

Results from the STRUCTURE and DAPC analyses suggest the current range of *B. affinis* is best described as 3 clusters delineated along an east-to-west gradient (“Twin Cities,” “Central,” and “Appalachian”; Fig. 2). Our sampling covers much of the extant range of the species with coverage within and among different USFWS Conservation Units (Fig. 1). Our analyses suggest that the northwest portion of the extant range (“Twin Cities”), which is within CU 1, is genetically differentiated from the rest of CU 1. CU 4, represented by the Appalachian population, represents a geographically disjunct population in the postdecline era, and it is also genetically disparate from the midwestern populations. What remains unknown is if this genetic structure developed due to the decline of *B. affinis* or if it represents long-standing genetic differentiation that predates the bee’s decline. Prior to our observations in *B. affinis*, no eastern North American bumble bee species exhibited substantial population structure across its range (Cameron et al 2011, Supplementary Fig. S1D therein), though population structure has been found in a western North American species complex, *B. bifarius* (Ghisbain et al. 2020). A possible explanation for the pattern of genetic differentiation is a truncation, due to regional extinction or severe genetic bottlenecks, of standing genetic variation structured continuously over space (Fig. 4). This possibility would anticipate intermediate genotypes to have existed in areas, such as Ohio, where *B. affinis* is now extinct and could be tested using historical museum specimens.

### Genetic Diversity

Analysis of genetic diversity among regional clusters did not reveal strong or consistent patterns across the range except to suggest that the Appalachian cluster appears to have different characteristics from the Central or Twin Cities clusters. The Appalachian cluster was typified by lower AR, but also a lower inbreeding coefficient (*F*_IS_; Fig. 3). The lower AR could be due to the smaller spatial extent of our samples (and the existing distribution of the species) within Virginia and West Virginia, thus providing fewer opportunities for rare alleles to be detected. Additionally, when compared to the 100-km population estimates of rarefied AR, the Appalachian specimens seem to be within the range observed across other putative populations (Fig. 3D). Although the Appalachian cluster appears distinct from other clusters, contextualizing the low AR within the spatial extent of collections, combined with the observation of low *F*_IS_ values compared to the other clusters (Fig. 3C), suggests that the Appalachian cluster does not have low genetic diversity. Instead, it appears to harbor a distinct set of alleles unobserved in other populations with high levels of private AR even when considering biases for sample size compared to other 100 km putative populations (Table 1). In comparison to Cameron et al. (2011), *H*_*e*_ values are within the range found from stable species in that study (range-wide mean of 0.678 in this study; range of 0.676–0.700 in Cameron et al. 2011 with at-risk species *B. pensylvanicus* and *B. occidentalis*, having 0.577 and 0.584, respectively). However, we found fairly high inbreeding coefficients (*F*_IS_) for many of the populations in this study (Fig. 3F) compared to values from other common or declining species (e.g., Dreier et al. 2014), suggesting that forces of inbreeding or other drivers of low heterozygosity, such as drift in small populations or recent bottlenecks, may be factors in the description of *B. affinis* genetics. However, caution needs to be taken when comparing heterozygosity values across studies, as different specimen collection processes, locus selection, scoring of alleles, or allelic composition may have a strong influence on those values (Bruford and Wayne 1993). Range wide, the values of AR and heterozygosity we observed overlap with values previously observed in both declining and stable species, suggesting that genetic diversity is within the range normally observed for bumble bee species.

We detected a range-wide diploid male frequency of ~15%; if looking only at populations with ≥10 males, the detected frequency is ~11% (Table 2). Compared to previous studies of male diploidy, these values do not appear to be outside of typical rates for stable species. Frequencies of male diploidy in the stable species, *B. vosnesenskii,* ranged from 0% to 86% in populations from coastal California (Schenau and Jha 2017). Other frequencies of 5% for *B. muscorum* in the United Kingdom (Darvill et al. 2006) and of *B. occidentalis* and *B. perplexus* at 6% and 3%, respectively, in Canada (Whidden and Owen 2011) have also been recorded. Although our observed frequency is higher, given the strength of our inference and the frequency observed, it would be difficult to infer this as substantially higher than previously reported values for these other species. Future studies that observe and model real frequencies of diploid males in multiple declining and stable bumble bee species would be useful to determine threshold levels indicative of the “diploid male vortex,” negative genetic-demographic feedback in hymenopterans (Zayed and Packer 2005, Leung and van der Meulen 2022).

### Genetic Differentiation

We find evidence for IBD, at least over fairly large geographic scales. This is consistent with prior studies of other North American bumble bee species (Lozier et al. 2011, Koch et al. 2017). Despite this trend, overall *F*_ST_ values are fairly low, suggesting that IBD occurs, but substantial gene flow may still happen across the species range. Alternatively, barriers to dispersal may be recent following the species’ decline, limiting current-day gene flow. Future research making direct comparisons to other species across similar landscapes may be necessary, but the geographically driven separation among populations lends support to the idea that separated populations of *B. affinis* represent distinct genetic units. More detailed studies of *B. affinis* dispersal among sites in relatively close proximity will be critical to determine the degree of gene flow among nearby sites and the ability of *B. affinis* to colonize nearby sites where it currently does not occur.

### Site-Level Colony Abundance

We find low colony abundance of *B. affinis* when compared to other species (Fig. 5). Even at sites with ≥9 specimens (relatively “strong” sites that represent persistent populations from year to year based on observations of local entomologists and site managers), colony abundance is lower than expected in comparison to other studies (Table 3) (e.g., Goulson et al. 2010, Mola et al. 2020). Overall, this suggests that *B. affinis* populations are typified by low colony abundance, even at sites with relatively high activity rates of observed workers. These low colony abundances suggest caution in activities that could remove or reduce the success of colonies. For example, the removal of only a single colony from a site would represent ~1.4%–70% of the colony abundance of our examined sites (Table 3). This information aids our understanding of some of the risks associated with a wide range of possible translocation activities (Smith et al. 2020). Likewise, this result suggests that, at most sites, population sizes are small enough that stochastic events could realistically cause local extinction of *B. affinis*. Understanding interannual variability in colony abundance would help to understand the risks of stochastic extinction events of these small populations and aid in determining the risk-benefit analysis associated with translocation interventions.

### Implications for Conservation and Management

The results of our study have several implications for conservation, management, and future research with *B. affinis* and likely other imperiled bumble bee species. The results of population structure analysis suggest that present-day regions of *B. affinis* may not be interchangeable. Although there is some caution warranted in interpreting these results due to the small spatial area of the Twin Cities and Appalachian clusters, these results suggest that management actions would do best to consider these populations on the end points of the known extant range individually and not as directly substitutable. Translocation methods are commonly considered in species recovery plans and include activities like reintroducing species to areas where they previously existed or supplementing extant populations with genetic material (i.e., reproductive males or females) from captively reared individuals (Ewen et al. 2012, Batson et al. 2015). These activities are under consideration for *B. affinis* (Smith et al. 2020). However, our results suggest some caution and further research is needed before population augmentation or genetic rescue should be implemented. For now, it seems prudent to view all 3 regions as distinct populations with potentially divergent management needs and, for the time being, to avoid moving genetic material among populations. The low colony abundance observed for *B. affinis* also has multiple implications for conservation and management. First, it suggests that even in areas deemed as strong-holds for *B. affinis*, colony abundance is far lower than expected in comparison to common or “stable” species, and so efforts to conserve the species may not only entail range expansion or management of areas where the species is infrequently detected but also consider actions that add to the density of *B. affinis* colonies at a location. Additionally, areas where *B. affinis* is infrequently or rarely detected may also be crucial to maintaining overall population connectivity. Second, any harvest of *B. affinis*, especially of queens, could have a substantial impact on those source populations. Before proceeding with captive rearing, surrogate species can be used to ensure that rearing facilities have high success rates, or at least better than expected rates in the wild. Our results show that the abundance of *B. affinis* colonies is low enough at sites for stochastic demographic processes to result in local extinctions. This suggests that there may be trade-offs in large-scale management actions, such as burning, mowing, and invasive species removal, that could benefit *B. affinis* habitats in the long run but increase the risk of stochastic extinction events in the short term. More research into each of these actions could thoroughly assess those risk-reward trade-offs.

### Conclusions

Rusty patched bumble bee (*B. affinis*) is now extirpated in ~70%–90% of its former range. Our study shows there is a current population structure in this species, roughly along an east–west gradient and with 3 major clusters, representing Minnesota, the rest of the upper Midwest, and Appalachia. At local scales, there is evidence for a loss of genetic diversity, and many population sizes, based on genetic mark-recapture, are very small compared to those of other bumble bee species where this has been quantified. These results are not entirely surprising for a species that has undergone a recent and rapid decline. However, further study is needed, especially in a comparative context with other co-occurring species or with more precise markers, as our results for heterozygosity and diploid male production are equivocal. Regardless, our results serve as a baseline for understanding the local and regional status of this species. Likewise, they point to clear information gaps about dispersal, demography, and genetic health where novel research may be of particular value. The conservation and recovery of *B. affinis* depends on population and genetic health (U.S. Fish and Wildlife Service 2021), and our study provides the first evidence defining those factors and providing a baseline for this endangered bumble bee.

## Supplementary Material

ieae041_suppl_Supplementary_Figures_S1

ieae041_suppl_Supplementary_Tables_S1

## References

[CIT0001] Adamack AT, Gruber B. PopGenReport: simplifying basic population genetic analyses in R. Methods Ecol Evol. 2014:5(4):384–387. 10.1111/2041-210x.12158

[CIT0002] Barton K, Barton MK. Package ‘mumin’. Version 1:439. 2015.

[CIT0003] Bates D, Mächler M, Bolker B, Walker S. Fitting linear mixed-effects models using lme4. J Stat Softw. 2015:67(1):1–48.

[CIT0004] Batson WG, Gordon IJ, Fletcher DB, Manning AD. REVIEW: translocation tactics: a framework to support the IUCN guidelines for wildlife translocations and improve the quality of applied methods. J Appl Ecol. 2015:52(6):1598–1607. 10.1111/1365-2664.12498

[CIT0005] Boone ML, Evans E, Wolf A, Minser H, Watson J, Smith TA. Notes from rusty patched bumble bee (*Bombus affinis* Cresson) nest observations. Insect Conserv Diver. 2022:15(3):380–384. 10.1111/icad.12564

[CIT0006] Bruford MW, Wayne RK. Microsatellites and their application to population genetic studies. Curr Opin Genet Dev. 1993:3(6):939–943. 10.1016/0959-437x(93)90017-j 8118220

[CIT0007] Cameron SA, Lozier JD, Strange JP, Koch JB, Cordes N, Solter LF, Griswold TL. Patterns of widespread decline in North American bumble bees. Proc Natl Acad Sci USA. 2011:108(2):662–667. 10.1073/pnas.1014743108 21199943 PMC3021065

[CIT0008] Cameron SA, Sadd BM. Global trends in bumble bee health. Annu Rev Entomol. 2020:65(1):209–232. 10.1146/annurev-ento-011118-111847 31610137

[CIT0009] Colla SR, Gadallah F, Richardson L, Wagner D, Gall L. Assessing declines of North American bumble bees (*Bombus* spp.) using museum specimens. Biodivers Conserv. 2012:21(14):3585–3595. 10.1007/s10531-012-0383-2

[CIT0010] Dakin EE, Avise JC. Microsatellite null alleles in parentage analysis. Heredity. 2004:93(5):504–509. 10.1038/sj.hdy.6800545 15292911

[CIT0011] Darvill B, Ellis JS, Lye GC, Goulson D. Population structure and inbreeding in a rare and declining bumblebee, *Bombus muscorum* (Hymenoptera: Apidae): inbreeding in a rare bumble bee. Mol Ecol. 2006:15(3):601–611. 10.1111/j.1365-294X.2006.02797.x 16499688

[CIT0012] Darvill B, Knight ME, Goulson D. Use of genetic markers to quantify bumblebee foraging range and nest density. Oikos. 2004:107(3):471–478. 10.1111/j.0030-1299.2004.13510.x

[CIT0013] Dreier S, Redhead JW, Warren IA, Bourke AFG, Heard MS, Jordan WC, Sumner S, Wang J, Carvell C. Fine-scale spatial genetic structure of common and declining bumble bees across an agricultural landscape. Mol Ecol. 2014:23(14):3384–3395. 10.1111/mec.12823 24980963 PMC4142012

[CIT0014] Estoup A, Garnery L, Solignac M, Cornuet J-M. Microsatellite variation in honey bee (*Apis mellifera* L.) populations: hierarchical genetic structure and test of the infinite allele and stepwise mutation models. Genetics. 1995:140(2):679–695. 10.1093/genetics/140.2.679 7498746 PMC1206644

[CIT0015] Estoup A, Solignac M, Cornuet JM, Goudet J, Scholl A. Genetic differentiation of continental and island populations of *Bombus terrestris* (Hymenoptera: Apidae) in Europe. Mol Ecol. 1996:5(1):19–31. 10.1111/j.1365-294x.1996.tb00288.x 9147693

[CIT0016] Evanno G, Regnaut S, Goudet J. Detecting the number of clusters of individuals using the software STRUCTURE: a simulation study. Mol Ecol. 2005:14(8):2611–2620. 10.1111/j.1365-294X.2005.02553.x 15969739

[CIT0017] Ewen JG, Armstrong DP, Parker KA, Seddon PJ. Reintroduction biology: integrating science and management. Oxford: John Wiley & Sons; 2012.

[CIT0018] Francis RM. pophelper: an R package and web app to analyse and visualize population structure. Mol Ecol Resour. 2017:17(1):27–32. 10.1111/1755-0998.12509 26850166

[CIT0019] Funk CR, Schmid‐Hempel R, Schmid‐Hempel P. Microsatellite loci for Bombus spp. Mol Ecol Notes. 2006:6(1):83–86.

[CIT0020] Funk WC, Forester BR, Converse SJ, Darst C, Morey S. Improving conservation policy with genomics: a guide to integrating adaptive potential into U.S. Endangered Species Act decisions for conservation practitioners and geneticists. Conserv Genet. 2019:20(1):115–134. 10.1007/s10592-018-1096-1

[CIT0021] Ghisbain G, Lozier JD, Rahman SR, Ezray BD, Tian L, Ulmer JM, Heraghty SD, Strange JP, Rasmont P, Hines HM. Substantial genetic divergence and lack of recent gene flow support cryptic speciation in a colour polymorphic bumble bee (*Bombus bifarius*) species complex. Syst Entomol. 2020:45(3):635–652. 10.1111/syen.12419

[CIT0022] Giles V, Ascher JS. A survey of the bees of the Black Rock Forest preserve, New York (Hymenoptera: Apoidea). J Hymenopt Res. 2006:15(2):208–231.

[CIT0023] Goudet J. Hierfstat, a package for R to compute and test hierarchical F-statistics. Mol Ecol Notes. 2005:5(1):184–186.

[CIT0024] Goulson D, Lepais O, O’Connor S, Osborne JL, Sanderson RA, Cussans J, Goffe L, Darvill B. Effects of land use at a landscape scale on bumblebee nest density and survival: landscape effects on bumblebee nest survival. J Appl Ecol. 2010:47(6):1207–1215. 10.1111/j.1365-2664.2010.01872.x

[CIT0025] Goulson D, Nicholls E. The canary in the coalmine; bee declines as an indicator of environmental health. Sci Prog. 2016:99(3):312–326. 10.3184/003685016X14685000479908 28742491 PMC10365390

[CIT0026] Hothorn T, Bretz F, Westfall P, Heiberger RM, Schuetzenmeister A, Scheibe S, Hothorn MT. Package ‘multcomp’. Simultaneous inference in general parametric models. Version 1.4.22. Vienna (Austria): Project for Statistical Computing; 2016.

[CIT0027] Jombart T. adegenet: a R package for the multivariate analysis of genetic markers. Bioinformatics. 2008:24(11):1403–1405. 10.1093/bioinformatics/btn129 18397895

[CIT0028] Jones OR, Wang J. COLONY: a program for parentage and sibship inference from multilocus genotype data. Mol Ecol Resour. 2010:10(3):551–555. 10.1111/j.1755-0998.2009.02787.x 21565056

[CIT0029] Kamvar ZN, Tabima JF, Grünwald NJ. Poppr: an R package for genetic analysis of populations with clonal, partially clonal, and/or sexual reproduction. PeerJ. 2014:2:e281. 10.7717/peerj.281 24688859 PMC3961149

[CIT0030] Kearse M, Moir R, Wilson A, Stones-Havas S, Cheung M, Sturrock S, Buxton S, Cooper A, Markowitz S, Duran C, et al. Geneious basic: an integrated and extendable desktop software platform for the organization and analysis of sequence data. Bioinformatics. 2012:28(12):1647–1649. 10.1093/bioinformatics/bts199 22543367 PMC3371832

[CIT0031] Koch JB, Looney C, Sheppard WS, Strange JP. Patterns of population genetic structure and diversity across bumble bee communities in the Pacific Northwest. Conserv Genet. 2017:18(3):507–520. 10.1007/s10592-017-0944-8

[CIT0032] Lepais O, Darvill B, O’Connor S, Osborne JL, Sanderson RA, Cussans J, Goffe L, Goulson D. Estimation of bumblebee queen dispersal distances using sibship reconstruction method. Mol Ecol. 2010:19(4):819–831. 10.1111/j.1365-294X.2009.04500.x 20089127

[CIT0033] Leung K, van der Meulen H. Revisiting the hymenopteran diploid male vortex: a review of avoidance mechanisms and incidence. Entomol Exp Appl. 2022:170(12):1010–1031.

[CIT0034] Lozier JD, Strange JP, Stewart IJ, Cameron SA. Patterns of range-wide genetic variation in six North American bumble bee (Apidae: *Bombus*) species. Mol Ecol. 2011:20(23):4870–4888. 10.1111/j.1365-294X.2011.05314.x 22035452

[CIT0035] Lozier JD, Zayed A. Bee conservation in the age of genomics. Conserv Genet. 2016:18(3):713–729. 10.1007/s10592-016-0893-7

[CIT0036] Mola JM, Miller MR, O’Rourke SM, Williams NM. Forests do not limit bumble bee foraging movements in a montane meadow complex. Ecol Entomol. 2020:45(5):955–965. 10.1111/een.12868

[CIT0037] Mola JM, Pearse I, Boone M, Evans E, Hepner M, Jean R, Kochanski J, Nordemeyer C, Runquist E, Smith TA, Strange J, Watson J, Koch J. Data from: Range-wide genetic analysis of an endangered bumble bee (*Bombus affinis*) reveals population structure, isolation by distance, and low colony abundance [Dataset]. . Dryad; 2024. 10.5061/dryad.8gtht76ws PMC10990054 38569059

[CIT0038] Mola JM, Stuligross C, Page ML, Rutkowski D, Williams NM. Impact of ‘non-lethal’ tarsal clipping on bumble bees (*Bombus vosnesenskii*) may depend on queen stage and worker size. J Insect Conserv. 2021:25(2):195–201. 10.1007/s10841-021-00297-9

[CIT0039] Mola JM, Williams NM. A review of methods for the study of bumble bee movement. Apidologie. 2019:50(4):497–514. 10.1007/s13592-019-00662-3

[CIT0040] Oksanen J, Simpson GL, Blanchet FG, Kindt R, Legendre P, Minchin PR, O’Hara RB, Solymos P, Stevens MHH, Szoecs E, et al. vegan: community ecology package. 2022. October 11.

[CIT0041] Owen RE, Whidden TL. Monandry and polyandry in three species of North American bumble bees (*Bombus*) determined using microsatellite DNA markers. Can J Zool. 2013:91(7):523–528. 10.1139/cjz-2012-0288

[CIT0042] Pompanon F, Bonin A, Bellemain E, Taberlet P. Genotyping errors: causes, consequences and solutions. Nat Rev Genet. 2005:6(11):847–859. 10.1038/nrg1707 16304600

[CIT0043] Pritchard JK, Stephens M, Donnelly P. Inference of population structure using multilocus genotype data. Genetics. 2000:155(2):945–959. 10.1093/genetics/155.2.945 10835412 PMC1461096

[CIT0044] R Core Team. R: a language and environment for statistical computing. Vienna (Austria): R Foundation for Statistical Computing; 2022.

[CIT0045] Schenau E, Jha S. High levels of male diploidy but low levels of genetic structure characterize *Bombus vosnesenskii* populations across the Western US. Conserv Genet. 2017:18(3):597–605. 10.1007/s10592-016-0900-z

[CIT0046] Smith TA, Strange JP, Evans EC, Sadd BM, Steiner JC, Mola JM, Traylor-Holzer K. Rusty patched bumble bee ex situ assessment and planning workshop: final report. Apple Valley (MN): IUCN SSC Conservation Planning Specialist Group; 2020. p. 85.

[CIT0047] Soro A, Quezada-Euan JJG, Theodorou P, Moritz RF, Paxton RJ. The population genetics of two orchid bees suggests high dispersal, low diploid male production and only an effect of island isolation in lowering genetic diversity. Conserv Genet. 2017:18:607–619.

[CIT0048] Stolle E, Rohde M, Vautrin D, Solignac M, Schmid-Hempel P, Schmid-Hempel R, Moritz RFA. Novel microsatellite DNA loci for *Bombus terrestris* (Linnaeus, 1758). Mol Ecol Resour. 2009:9(5):1345–1352. 10.1111/j.1755-0998.2009.02610.x 21564905

[CIT0049] Szymanski J, Smith T, Horton A, Parkin M, Ragan L, Masson G, Olson E, Gifford K, Hill L. Rusty patched bumble bee (*Bombus affinis*) species status assessment final report. version 1. June. 2016.

[CIT0050] U.S. Fish and Wildlife Service. Final rule: endangered species status for rusty patched bumble bee. Fed Regist. 2017a:82:3186–3209.

[CIT0051] U.S. Fish and Wildlife Service. Final rule; delay of effective date. Endangered and threatened wildlife and plants: endangered species status for rusty patched bumble bee. Fed Regist. 2017b:82:10285–10286.

[CIT0052] U.S. Fish and Wildlife Service. Recovery plan for rusty patched bumble bee (*Bombus affinis*). Bloomington (MN): Midwest Regional Office; 2021.

[CIT0053] Wagner DL. Insect declines in the Anthropocene. Annu Rev Entomol. 2020:65:457–480. 10.1146/annurev-ento-011019-025151 31610138

[CIT0054] Wang J. A new method for estimating effective population sizes from a single sample of multilocus genotypes. Mol Ecol. 2009:18(10):2148–2164. 10.1111/j.1365-294X.2009.04175.x 19389175

[CIT0055] Weir BS, Cockerham CC. Estimating *F*-statistics for the analysis of population structure. Evolution. 1984:38(6):1358–1370. 10.1111/j.1558-5646.1984.tb05657.x 28563791

[CIT0056] Whidden TL, Owen RE. Frequencies of diploid males in natural populations of three north American bumble bee (*Bombus*) species (Hymenoptera: Apidae). Ann Entomol Soc Am. 2011:104(1):83–87. 10.1603/an10092

[CIT0057] Zayed A. Effective population size in Hymenoptera with complementary sex determination. Heredity. 2004:93(6):627–630. 10.1038/sj.hdy.6800588 15354193

[CIT0058] Zayed A, Packer L. Complementary sex determination substantially increases extinction proneness of haplodiploid populations. Proc Natl Acad Sci USA. 2005:102(30):10742–10746. 10.1073/pnas.0502271102 16020532 PMC1180771

[CIT0059] Zayed A, Roubik DW, Packer L. Use of diploid male frequency data as an indicator of pollinator decline. Proc R Soc B Suppl. 2003:271(Suppl 3):S1–S4. 10.1098/rsbl.2003.0109 PMC1809989 15101404

