# Supplementary material for: Range-wide genetic analysis of an endangered bumble bee (Bombus affinis, Hymenoptera: Apidae) reveals population structure, isolation by distance, and low colony abundance
Source: J Insect Sci. 2024 Apr 3;24(2):19. doi: 10.1093/jisesa/ieae041 (PMC10990054; doi:10.1093/jisesa/ieae041)

**Supplemental Figure S1:** Map of the 59 sites of *Bombus affinis* identified using the 10-km clustering procedure as described in the main text. Points are scaled by number of samples from that site. Color of the points represents the 100-km putative populations of *Bombus affinis* and matches the color of individual collections displayed in main text Figure 1. States are colored by their Conservation Unit, with CU 5 not displayed for visual clarity as there are no contemporary observations nor is CU 5 bordered by units with observations.


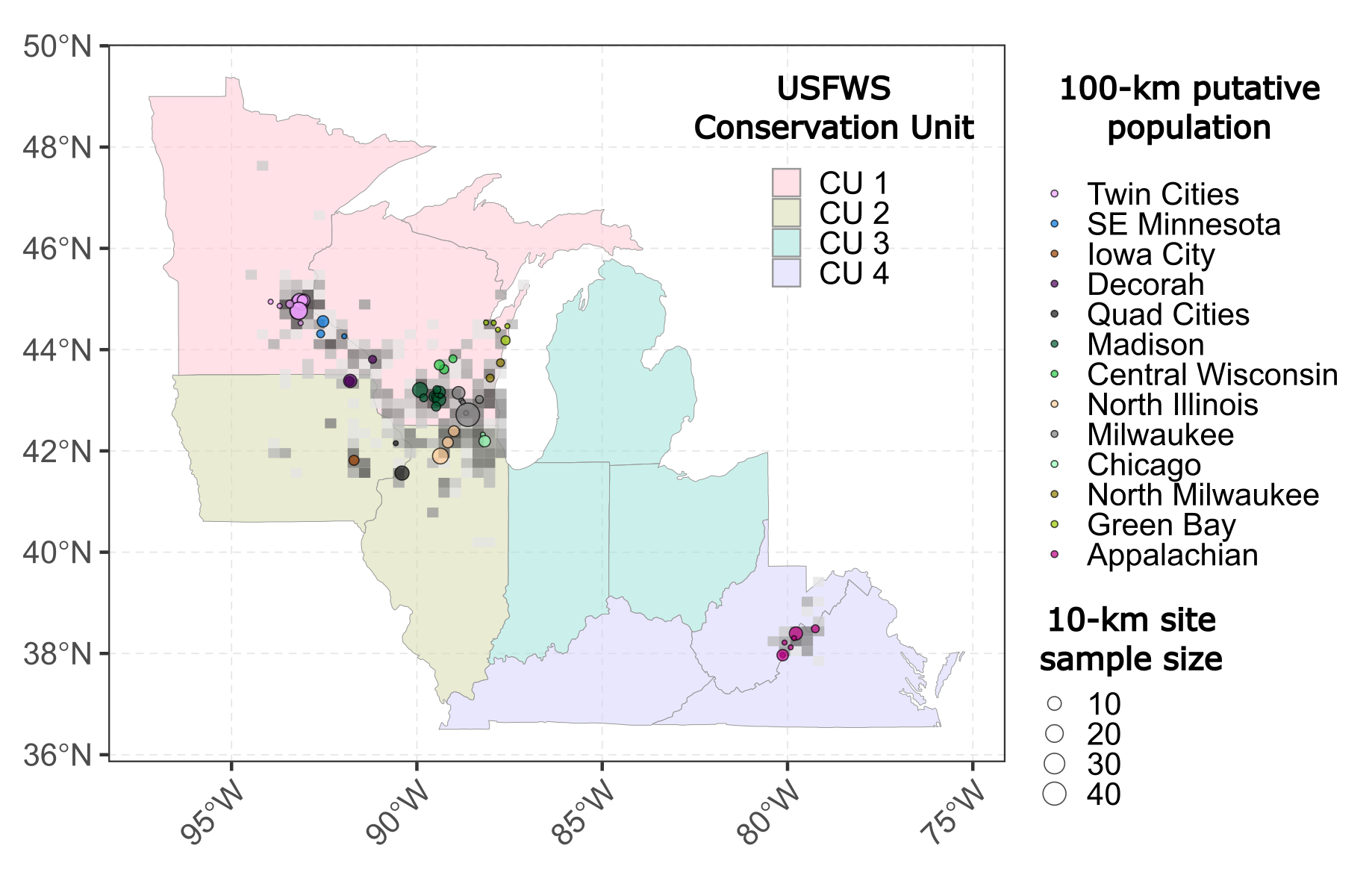

Supplement: ieae041_suppl_Supplementary_Figures_S1 [file ieae041_suppl_supplementary_figures_s1.docx]
